# Genome Sequences of *Gordonia* Bacteriophages Obliviate, UmaThurman, and Guacamole

**DOI:** 10.1128/genomeA.00595-16

**Published:** 2016-06-30

**Authors:** Welkin H. Pope, Armaan F. Akbar, Taylor N. Ayers, Selena G. Belohoubek, Connie F. Chung, Allison C. Hartman, Tejus Kayiti, Cecilia M. Kessler, Philipp I. Koman, Grigoriy A. Kotovskiy, Taylor M. Morgan, Rebecca M. Rohac, Gabriela M. Silva, Charles E. Willis, Katherine A. Milliken, Kathleen A. Shedlock, Ann-Catherine J. Stanton, Chelsea L. Toner, Emily C. Furbee, Sarah R. Grubb, Marcie H. Warner, Matthew T. Montgomery, Rebecca A. Garlena, Daniel A. Russell, Deborah Jacobs-Sera, Graham F. Hatfull

**Affiliations:** Department of Biological Sciences, University of Pittsburgh, Pittsburgh, Pennsylvania, USA

## Abstract

We describe three newly isolated phages—Obliviate, UmaThurman, and Guacamole—that infect *Gordonia terrae* 3612. The three genomes are related to one another but are not closely related to other previously sequenced phages or prophages. The three phages are predicted to use integration-dependent immunity systems as described in several mycobacteriophages.

## GENOME ANNOUNCEMENT

*Gordonia terrae* 3612 and *Mycobacterium smegmatis* are both members of the taxonomic order *Corynebacteriales*. The hundreds of sequenced mycobacteriophages display considerable genetic diversity (1), but few *Gordonia* phage genome sequences are available, and their diversity and relationships to the mycobacteriophages are ill defined (2–7). Isolation and characterization of *Gordonia* phages in the Science Education Alliance–Phage Hunters Advancing Genomics and Evolutionary Science (SEA-PHAGES) program assists in addressing these questions (1, 8).

Phages Obliviate, UmaThurman, and Guacamole were isolated by direct plating of soil samples from Pittsburgh, Pennsylvania, USA, on lawns of *G. terrae* 3612. They were then plaque-purified and amplified, and their DNA was extracted. All three phages have similar virion morphologies with 50-nm diameter isometric heads and long flexible tails, approximately 250 nm long. Each genome was sequenced using the Illumina MiSeq platform, and 140-bp single-end reads were assembled into major single contigs with lengths of 49,286 bp, 50,127 bp, and 49,894 bp with 619-fold, 1,434-fold, and 809-fold coverage for Obliviate, UmaThurman, and Guacamole, respectively. All have defined ends with 10-base 3′ extensions (Obliviate and Guacamole: 5′-TCGCCGGTGA; UmaThurman: 5′-TCTCCGGTGA). The GC contents of the genomes are 67.2, 67.5, and 67.0%, similar to *G. terrae* (67.8%). The three phages share extensive nucleotide sequence similarity with pairwise 96% nucleotide sequence identity spanning 72 to 82% of their genome lengths. The greatest similarities are within the virion structural and assembly genes, with interspersed segments of similarity within the nonstructural genes. The phages do not share extensive nucleotide sequence similarity with other phages or predicted prophages, although there are two small segments (~1.5 kb) with similarity to putative capsid assembly protease and lysis genes of a potential prophage in *Gordonia* sp. KTR9 (9).

Using GeneMark and GLIMMER (10, 11) we identified 80, 83, and 78 protein-coding genes in Obliviate, UmaThurman, and Guacamole, respectively; none of the genomes encode tRNAs.

All the predicted genes are transcribed rightward, with the exception of five to seven leftward-transcribed genes near the genome centers that include putative immunity repressors and integrase genes. The genome left arms contain the virion structure and assembly genes, and the right arms contain nonstructural genes, including *recET* recombinases. The lysis genes are unusually located amid the phage tail gene cluster, and two genes, Obliviate *19* and *20*, encode endolysin functions—the peptidase and glycoside hydrolase domains, respectively.

Obliviate is predicted to use an integration-dependent immunity system as described for some mycobacteriophages (12, 13), characterized by the location of the phage attachment site (*attP*) within the repressor gene (*38*), and a degradation tag (-DAA) at the C-terminus of gp38. Obliviate is predicted to integrate at an *attB* site overlapping a *Gordonia* tRNA^thr^ gene (KRT9_RS04270). Guacamole encodes a distantly related repressor (gp40; 41% amino acid [aa] identity with Obliviate gp38), although it contains the same *attP* core and is predicted to integrate at the same *attB* site. The UmaThurman repressor (gp36) is more distantly related to the Obliviate/Guacamole repressors (<40% aa identity) and contains a different *attP* corresponding to an *attB* site overlapping a tRNA^arg^ gene conserved in many actinobacterial strains.

### Nucleotide sequence accession numbers.

The Obliviate, UmaThurman, and Guacamole genomes are available from GenBank under the accession numbers KU963254, KU963251, and KU963259.
